# Recurrent Bilateral Occipital Infarct with Cortical Blindness and Anton Syndrome

**DOI:** 10.1155/2014/795837

**Published:** 2014-03-13

**Authors:** Kiu Kwong Yew, Sanihah Abdul halim, Ahmad Tajudin Liza-Sharmini, John Tharakan

**Affiliations:** ^1^Department of Ophthalmology, School of Medical Sciences, Universiti Sains Malaysia, Health Campus, 16150 Kubang Kerian, Kelantan, Malaysia; ^2^Department of Medicine, School of Medical Sciences, Universiti Sains Malaysia, Health Campus, 16150 Kubang Kerian, Kelantan, Malaysia; ^3^Department of Neuroscience, School of Medical Sciences, Universiti Sains Malaysia, Health Campus, 16150 Kubang Kerian, Kelantan, Malaysia

## Abstract

Bilateral cortical blindness and Anton syndrome, are most commonly caused by ischaemic stroke. In this condition, patients have loss of vision but deny their blindness despite objective evidence of visual loss. We report a case of a patient with multiple cardiovascular risk factors who developed recurrent bilateral occipital lobe infarct with Anton syndrome. A suspicion of this condition should be raised when the patient has denial of blindness in the presence of clinical and radiological evidence of occipital lobe injury. Management of this condition should focus on the underlying cause, in which our patient requires secondary stroke prevention and rehabilitation.

## 1. Introduction

Cortical blindness refers to loss of vision caused by bilateral occipital lobe lesions with presence of intact anterior visual pathway [1, 2]. Anton syndrome (visual anosognosia) is a rare complication of cortical blindness with denial of loss of vision by patient who is unable to see [2, 3]. Such patient may confabulate during visual examinations or offer excuses for their symptoms or may endanger themselves to prove that they are capable of seeing [2]. With damage to the visual association cortex, patients are unable to acknowledge their visual deficit [2, 4]. Ischemic stroke is the most common cause of cortical blindness [1, 4]. We describe one case with Anton syndrome secondary to recurrent bilateral occipital infarct.

## 2. Case Presentation 

A 57-year-old man with background history of diabetes mellitus, hypertension, hyperlipidemia, and bilateral occipital lobe infarct 5 years ago presented with sudden bilateral loss of vision for a 3-day duration associated with slurred speech. It was preceded by occipital headache.

He had history of bilateral occipital lobe infarcts five years ago with both eyes (OU) vision of only perception to light (PL). There was no neurological deficit apart from slurring of speech. He had no symptoms of denial of visual deficit at that time. CT brain showed multiple infarcts in both parietooccipital regions. One month after being discharged from hospital, his vision still maintained at PL OU and it slowly recovered. He was capable of watching television and reading with glasses 6 months after the stroke. Since then, the vision remained stable until the current events. His vision prior to the first episode of stroke was clear without glasses. He was not compliant to his medication.

**Figure 1:**
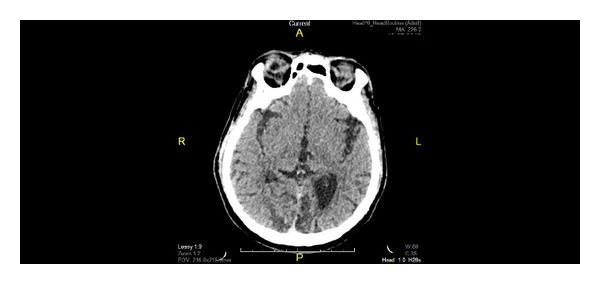
Noncontrasted CT brain shows bilateral occipital hypodense lesion with dilated left lateral ventricles.

On arrival in emergency unit during this episode, he was fully conscious with blood pressure of 124/83 mmHg. He was orientated to time, place, and person. Apart from slurred speech, he had normal power in all four limbs and intact sensation. He had severe visual impairment with hand motion OU. Pupils were reactive; corneal reflexes were intact with normal fundoscopic findings. There was no blink response. A CT brain (Figure 1) showed bilateral occipital lobe infarcts with dilated left lateral ventricles. In the ward, he claimed he can see but was unable to name the objects shown to him. He claimed he can see the floor but was not sure about the colour. He walked with support and claimed his body was weak rather than loss of vision. Visual evoked potential (VEP) was done and revealed absence of input potential. He was started on secondary stroke prevention medications. One week later, he was discharged with OU vision of hand motion. Upon discharge, he did not deny visual deficit anymore.

## 3. Discussion 

Cortical blindness with Anton syndrome (visual anosognosia) is characterized by denial of blindness by patient who is unable to see in the presence of intact anterior visual pathways. Several case reports had been founded to describe the disease associated with cerebral vascular accident, obstetric hemorrhage, and advanced glaucoma [1, 2, 5, 6]. Neurological visual impairment as a result of brain damage encompasses a broad spectrum of manifestations such as cortical blindness, visual neglect, visual agnosia, denial of blindness homonymous hemianopia, lack of facial recognition, and delayed visual development [3]. The characteristic of cortical blindness includes (i) loss of visual sensations, (ii) loss of menace reflex, (iii) preservation of light and accommodation pupillary reflexes, (iv) a normal fundus, and (v) preservation of ocular movement [7].

Anton syndrome is usually associated with bilateral occipital infarcts where it is supplied by the posterior cerebral arteries and these infarcts usually involve both the primary visual cortex and visual association area. Area of parietal and temporal lobes can be involved as well [8]. The occipital cortex is sensitive to systemic hypoxia due to its relatively distal location from the central cerebral vasculature [9]. There are few explanations postulated for visual anosognosia. First, denial of blindness could be related to memory loss or confusion. Second, the visual monitor, which is one of the visual association areas, might have been damaged. Normally, the visual monitor assesses the input and provides other parts of the brain with information such as speech area. When the visual monitor is destroyed or disconnectedfrom the speech area, absence of input makes the patient confabulate a response. A third mechanism could be due to false feedback to visual association area which is linked by second visual system mediated by superior colliculus, pulvinar, and temporoparietal regions [6, 8].

The prognosis for patients with cortical blindness depends on the age, medical history, cause, severity, and duration as well as the speed of initial recovery [1, 2, 5]. Good recovery of visual function has been noted in conditions such as hypertensive encephalopathy, cardiac surgery, cerebral angiography, and infective endocarditis [1, 3, 10]. Aldrich et al. [1] mentioned that better visual outcome was observed in (i) young patient (<40 years old), (ii) no history of hypertension and diabetes, (iii) no cognitive, language, or memory impairment, and (iv) CVA is not the causative factor.

As in our patient, he had two episodes of stroke events, but he was only being noticed to have denial of blindness in the second episode. This patient had recovered with good vision after few months of the 1st attack. However, most of the cortical blindness cases associated with stroke had reported poor visual recovery. In view of multiple risk factors for stroke and noncompliance to medication, the recurrence risk is high for him. The speed of recovery and visual improvement might be slow after the second attack due to additional factor mentioned above. Nonetheless, long-term followup is needed to assess the final visual outcome. There are few cases that have been reported as cortical blindness with Anton syndrome secondary to cerebrovascular accident. Perhaps this is the first time we founded Anton syndrome associated with recurrent bilateral occipital infarct.

In 1895, the Australian neuropsychiatrist Gabriel Anton (1858–1933) described a case of a 69-year-old dairymaid who had blindness and deafness with lack of self-perception of the deficits. He associated these with lesion on her both temporal lobes. In 1914, the French-Polish neurologist Joseph Francois Babinski (1857–1932) used the term “anosognosia” to describe the unawareness of the deficit in patients with hemiplegia [3, 4]. CT brain is helpful when there is presence of low attenuation areas in the occipital lobes or cerebral oedema which supports the diagnosis of cortical blindness and assists in exclusion of hemorrhage or neoplastic etiology. MRI is recommended in some literature as the diagnostic imaging technique of choice. Benefits of MRI include superior detection of subtle vasogenic oedema as well as detailed evaluation of the venous sinuses and anterior visual tracts [2, 5]. The role of EEG and VEP in evaluating cortical blindness and its prognostic value remains controversial [1].

## 4. Conclusion

A suspicion of cortical blindness and Anton syndrome (visual anosognosia) should be raised when the patient has denial of blindness with evidence of occipital lobe injury. Our case adds on limited reference for Anton syndrome. Management of this condition should emphasise on the secondary prevention and rehabilitation.
